# Prognostic nomogram for patients with unresectable pancreatic cancer treated with gemcitabine plus nab–paclitaxel or FOLFIRINOX: A post–hoc analysis of a multicenter retrospective study in Japan (NAPOLEON study)

**DOI:** 10.1186/s12885-021-09139-y

**Published:** 2022-01-03

**Authors:** Taro Shibuki, Toshihiko Mizuta, Mototsugu Shimokawa, Futa Koga, Yujiro Ueda, Junichi Nakazawa, Azusa Komori, Satoshi Otsu, Shiho Arima, Masaru Fukahori, Akitaka Makiyama, Hiroki Taguchi, Takuya Honda, Kenji Mitsugi, Kenta Nio, Yasushi Ide, Norio Ureshino, Tsuyoshi Shirakawa, Taiga Otsuka

**Affiliations:** 1 Department of Internal Medicine, Imari Arita Kyoritsu Hospital, 860 Ninose-ko, Arita-cho, Nishi-matsuura-gun, Saga, 849-4193 Japan; 2 Department of Hepatobiliary and Pancreatic Oncology, National Cancer Center Hospital East, 6-5-1 Kashiwanoha, Kashiwa-shi, Chiba, 277-8577 Japan; 3 Department of Internal Medicine, Fujikawa Hospital, 1-2-6 Matsubara, Saga-shi, Saga, 840-0831 Japan; 4 Clinical Research Institute, National Hospital Organization Kyushu Cancer Center, 3-1-1 Notame, Minami-ku, Fukuoka-shi, Fukuoka, 811-1395 Japan; 5 Department of Biostatistics, Yamaguchi University Graduate School of Medicine, 1-1-1 Minamikogushi, Ube, Yamaguchi, 755-8505 Japan; 6 Department of Hepatobiliary and Pancreatology, Saga Medical Center Koseikan, 400 Kase-machi, Saga-shi, Saga, 840-8571 Japan; 7 Department of Hematology and Oncology, Japanese Red Cross Kumamoto Hospital, 2-1-1 Nagamine-minami, Higashi-ku, Kumamoto-shi, Kumamoto, 861-8520 Japan; 8 Department of Medical Oncology, Kagoshima City Hospital, 37-1 Uearata-cho, Kagoshima-shi, Kagoshima, 890-8760 Japan; 9 Department of Medical Oncology and Hematology, Oita University Faculty of Medicine, 1-1 Idaigaoka, Hasama-machi, Yufu-shi, Oita 879-5593 Japan; 10 Digestive and Lifestyle Diseases, Kagoshima University Graduate School of Medical and Dental Sciences, 8-35-1 Sakuragaoka, Kagoshima-shi, Kagoshima, 890-8520 Japan; 11 Division of Gastroenterology, Department of Medicine, Kurume University School of Medicine, 67 Asahi-machi, Kurume-shi, Fukuoka, 830-0011 Japan; 12 Department of Hematology/Oncology, Japan Community Healthcare Organization Kyushu Hospital, 1-8-1 Kishinoura, Yahatanishi-ku, Kitakyushu-shi, Fukuoka, 806-8501 Japan; 13 Cancer Center, Gifu University Hospital, 1-1 Yanagido, Gifu-shi, Gifu, 501-1194 Japan; 14 Department of Gastroenterology, Saiseikai Sendai Hospital, 2-46 Harada-machi, Satsumasendai-shi, Kagoshima, 895-0074 Japan; 15 Department of Gastroenterology, Izumi General Medical Center, 520 Myojincho, Izumi-shi, Kagoshima, 899-0131 Japan; 16 Department of Gastroenterology and Hepatology, Nagasaki University Graduate School of Biomedical Sciences, 1-7-1 Sakamoto, Nagasaki-shi, Nagasaki, 852-8501 Japan; 17 Department of Medical Oncology, Hamanomachi Hospital, 3-3-1 Nagahama, Chuo-ku, Fukuoka-shi, Fukuoka, 810-8539 Japan; 18 Department of Medical Oncology, Sasebo Kyosai Hospital, 10-17 Shimanji-cho, Sasebo-shi, Nagasaki, 857-8575 Japan; 19 Department of Internal Medicine, Karatsu Red Cross Hospital, 2430 Watada, Karatsu-shi, Saga, 847-8588 Japan; 20 Department of Medical Oncology, Saga Medical Center Koseikan, 400 Kase-machi, Saga-shi, Saga, 840-8571 Japan; 21 Department of Medical Oncology, Kimitsu Chuo Hospital, 1010 Sakurai, Kisarazu-shi, Chiba, 292-8535 Japan; 22 Department of Medical Oncology, Fukuoka Wajiro Hospital, 2-2-75 Wajirogaoka, Higashi-ku, Fukuoka-shi, Fukuoka, 811-0213 Japan; 23 Karatsu Higashi-matsuura Medical Association Center, 2566-11 Chiyoda-machi, Karatsu-shi, Saga, 847-0041 Japan; 24 Department of Internal Medicine, Minato Medical Clinic, 3-11-3 Nagahama, Chuo-ku, Fukuoka-shi, Fukuoka, 810-0072 Japan

**Keywords:** unresectable pancreatic cancer, nomogram, prognosis, overall survival, chemotherapy

## Abstract

**Background:**

No reliable nomogram has been developed until date for predicting the survival in patients with unresectable pancreatic cancer undergoing treatment with gemcitabine plus nab–paclitaxel (GnP) or FOLFIRINOX.

**Methods:**

This analysis was conducted using clinical data of Japanese patients with unresectable pancreatic cancer undergoing GnP or FOLFIRINOX treatment obtained from a multicenter study (NAPOLEON study). A Cox proportional hazards model was used to identify the independent prognostic factors. A nomogram to predict 6–, 12–, and 18–month survival probabilities was generated, validated by using the concordance index (C–index), and calibrated by the bootstrapping method. And then, we attempted risk stratification for survival by classifying the patients according to the sum of the scores on the nomogram (total nomogram points).

**Results:**

A total of 318 patients were enrolled. A prognostic nomogram was generated using data on the Eastern Cooperative Oncology Group performance status, liver metastasis, serum LDH, serum CRP, and serum CA19–9. The C–indexes of the nomogram were 0.77, 0.72 and 0.70 for 6–, 12–, and 18–month survival, respectively. The calibration plot showed optimal agreement at all points. Risk stratification based on tertiles of the total nomogram points yielded clear separations of the survival curves. The median survival times in the low–, moderate–, and high–risk groups were 15.8, 12.8 and 7.8 months (*P*<0.05), respectively.

**Conclusions:**

Our nomogram might be a convenient and inexpensive tool to accurately predict survival in Japanese patients with unresectable pancreatic cancer undergoing treatment with GnP or FOLFIRINOX, and will help clinicians in selecting appropriate therapeutic strategies for individualized management.

**Supplementary Information:**

The online version contains supplementary material available at 10.1186/s12885-021-09139-y.

## Background

Pancreatic cancer is the seventh leading cause of cancer–related death worldwide, and the fourth leading cause of cancer death in Japan [1, 2]. Although surgical resection is the only curative treatment for pancreatic cancer, only 15% of pancreatic cancer patients are suitable candidates for curative pancreatectomy, because most patients have either distant metastases or locoregional spread, including vascular invasion, even at diagnosis [3]. Palliative chemotherapy is used for patients diagnosed as having unresectable pancreatic cancer. Recently, great strides have been made in palliative chemotherapy for patients with metastatic pancreatic cancer due to development of the gemcitabine plus nab–paclitaxel (GnP) and FOLFIRINOX (fluorouracil, leucovorin, irinotecan, and oxaliplatin) regimens [4, 5]. However, the overall prognosis of pancreatic cancer remains unsatisfactory. The 5–year survival of patients with pancreatic cancer is a dismal 8% [6]. One of the reasons for the high mortality rate of pancreatic cancer patients may be the absence of a reliable method for prognosis determination and risk stratification. If the prognosis of pancreatic cancer patients could be evaluated more accurately, we could offer better therapeutic strategies and individualized treatments.

The American Joint Committee on Cancer (AJCC) TNM staging system, which is based on the tumor characteristics, and presence/absence of nodal and distant metastases, is currently the mainly used system to predict survival in patients with cancers, including pancreatic cancer [7, 8]. Because patients with unresectable pancreatic cancer would be roughly classifiable as stage III or IV, the AJCC staging system is relatively difficult to discriminate for prediction of survival even in patients with the same AJCC stage [8]. Furthermore, it should be recognized that the AJCC TNM staging system only takes into account three tumor–related factors and not patient–specific factors such as the age, sex, race, and marital status, all of which are known to be associated with the survival in pancreatic cancer patients [7]. Therefore, an individualized, more accurate prognostic system is desirable.

A nomogram is a scoring and visualization tool of a multivariate predictive model, and is accepted as a reliable scale for more accurate survival prediction in individual patients as compared to the AJCC staging system [9–12]. However, to the best of our knowledge, no reliable nomogram has been developed yet for predicting survival in patients with unresectable pancreatic cancer undergoing treatment with GnP or FOLFIRINOX, which is currently recognized as the standard chemotherapy for these patients. In the present study, we attempted to develop a prognostic nomogram for patients with unresectable pancreatic cancer receiving GnP or FOLFIRINOX treatment, based on the real–world data.

## Methods

### Patients

This was a multicenter retrospective study of patients with unresectable or recurrent pancreatic cancer who underwent treatment with GnP or FOLFIRINOX at any of 14 centers in Kyushu, Japan (NAPOLEON study). We retrospectively reviewed the hospital medical records of the patients for the period between December 2013 and March 2017, and consecutive patients with locally advanced or metastatic pancreatic cancer were included. The following variables of the patients were investigated: the patient demographic characteristics (age, sex and body mass index), Eastern Cooperative Oncology Group performance status (ECOG PS), history of previous therapy (tumor resection and adjuvant chemotherapy), tumor size, tumor location (pancreatic head, body, or tail), tumor histology (adenocarcinoma, or not), sites of metastasis (liver, peritoneum, and/or lung), number of metastatic sites (one, or two or more), presence/absence of ascites, the AJCC TNM stage, and serum albumin, lactate dehydrogenase (LDH), C–reactive protein (CRP), carcinoembryonic antigen (CEA) and carbohydrate antigen 19–9 (CA19–9) levels, and the first–line chemotherapy regimen used (GnP or FOLFIRINOX). These data were collected by clinicians with expertise in clinical research under the supervision of the statistician and centrally managed. This study was conducted with the approval of the institutional review board of each participating institution, and according to the principles laid down in the Declaration of Helsinki.

### Treatment

All patients received either GnP or FOLFIRINOX as the first–line regimen. The GnP group consisted of patients who received nab–paclitaxel at the dose of 125 mg/m^2^ given as a 30–minute intravenous infusion, followed by GEM at the dose of 1000 mg/m^2^ given as a 30–minute intravenous infusion on days 1, 8, and 15, every 4 weeks [4]. The FOLFIRINOX group received either the original or the modified regimen. The original FOLFIRINOX regimen consists of a combination of oxaliplatin at the dose of 85 mg/m^2^ given as a 2–hour intravenous infusion, followed by l–leucovorin at the dose of 200 mg/m^2^ given as a 2–hour intravenous infusion, with the addition, after 30 minutes, of irinotecan at the dose of 180 mg/m^2^ given as a 90–minute intravenous infusion, followed by fluorouracil at the dose of 400 mg/m^2^ given as an intravenous bolus injection, followed by a continuous intravenous fluorouracil infusion at 2400 mg/m^2^ over a 46–hour period, every 2 weeks. The modified FOLFIRINOX regimen consists of a combination of oxaliplatin at the dose of 85 mg/m^2^ given as a 2–hour intravenous infusion, followed by l–leucovorin at the dose of 200 mg/m^2^ given as a 2–hour intravenous infusion, with the addition, after 30 minutes, of irinotecan at the dose of 150 mg/m^2^ given as a 90–minute intravenous infusion, followed by continuous intravenous fluorouracil infusion at 2400 mg/m^2^ over a 46–hour period, every 2 weeks [13, 14].

### Assessments

The goal of this study was to identify factors influencing the prognosis in pancreatic cancer patients, and then to develop and validate a prognostic nomogram in a relatively large real–world cohort derived from the NAPOLEON study. The overall survival was calculated as the interval from the date of initiation of first–line chemotherapy to the date of death from any cause or the date of the last follow–up. The 8^th^ edition of the AJCC staging system for pancreatic cancer was used [15]. The pancreatic cancer stages are categorized as follows: Stage I A, T1 N0 M0; Stage I B, T2 N0 M0; Stage II A, T3 N0 M0; Stage IIB, T1–3 N1 M0; Stage III, T–Any N2 M0 or T4 N–Any M0; and Stage IV, T– Any N–Any M1.

### Statistical analysis and drawing of the nomogram

**Fig. 1:**
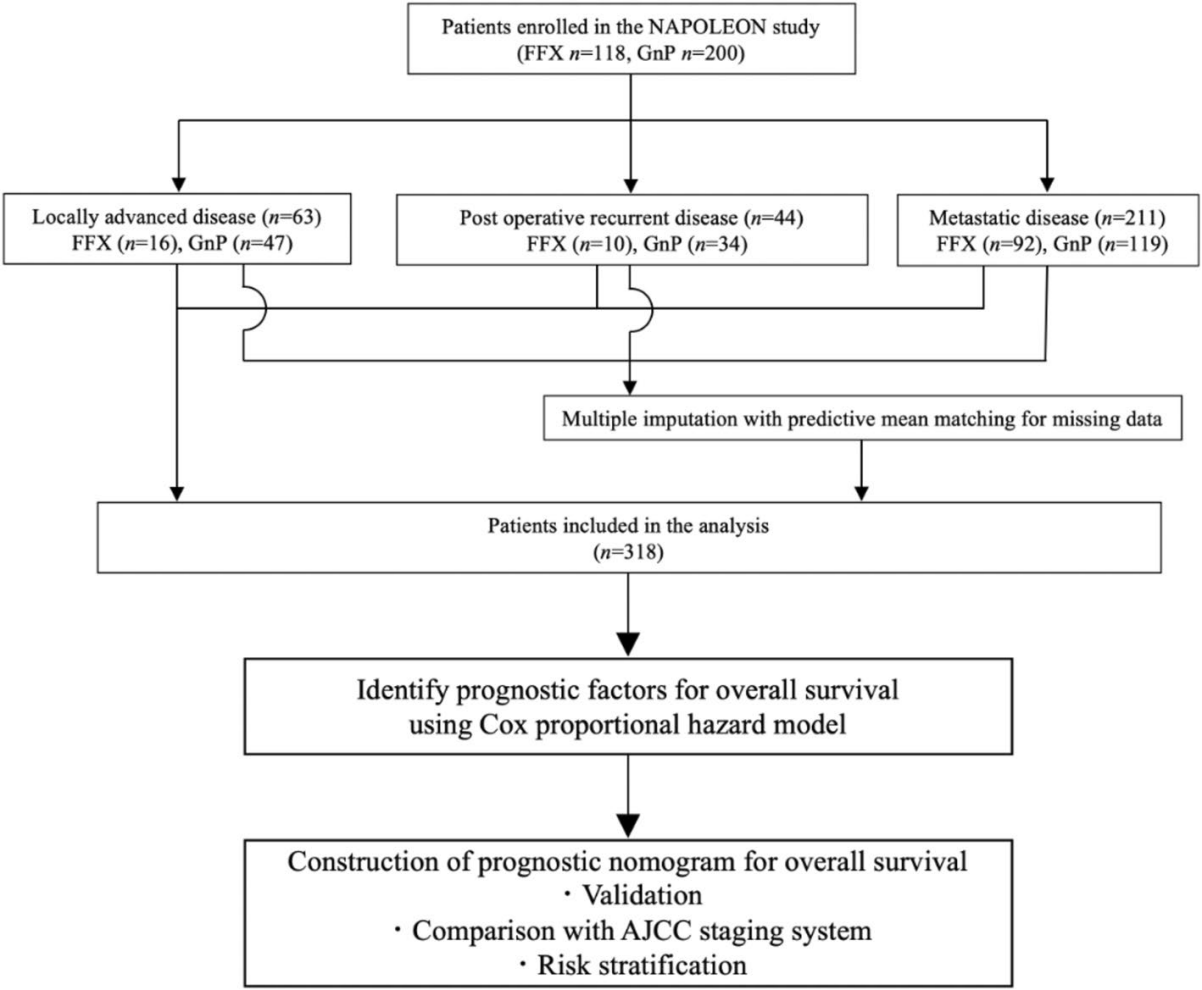
Flow diagram showing the development of the prognostic nomogram.

Missing data were imputed by using the method of multiple imputation with predictive mean matching [16]. The imputation model included variables for tumor size, and serum albumin, LDH, CRP and CA19–9 levels. The Cox proportional hazard model was used to identify the independent prognostic factors for overall survival. Factors showing differences with *P* values of <0.05 were considered as being statistically significant. Prognostic factors judged to be clinically important and those with *P* values of <0.05 were selected, and a prognostic nomogram to predict the 6–, 12–, and 18–month survival probabilities was generated on the basis of the final model and validated using the concordance index (C–index) and calibration plot by the bootstrapping method (200 resamplings). The final model was compared with the AJCC TNM staging system to assess discrimination ability for survival prediction based on the time–dependent area under the curve (*t*–AUC) in a Receiver Operating Characteristic (ROC) curve analysis. And then, we attempted to develop a method of risk stratification for survival according to the tertiles of the total nomogram points and compared the survival times among the risk groups (Fig. 1). The advantage of this nomogram over the AJCC TNM staging system in predicting survival was confirmed to compare the C–indexes of the nomogram and AJCC TNM staging system. The statistical analyses were performed using the software program R ver. 3.6.1 (R Foundation for Statistical Computing, Vienna, Austria).

## Results

A total of 318 patients were enrolled between December 2013 and March 2017. The baseline characteristics of the 318 patients are shown in Table 1. By the end of the follow–up period, 197 patients (61.9%) had died. Of the 197 deaths, 195 patients died from pancreatic cancer and 2 patients were died from other diseases. The median overall survival was 11.3 months, and the median follow-up period was 11.4 months.

**Table 1:** Baseline characteristics

	*n*=318
Age (years), *n* (%)	
<60	93 (29)
60 – 70	137 (43)
>70	88 (28)
Sex (M/F), *n* (%)	196/122 (62/38)
ECOG PS, *n* (%)	
0	204 (64)
1	96 (30)
2 or more	17 (5)
Body mass index (kg/m^2^), *n* (%)	
<22	198 (62)
≥22	120 (38)
Tumor resection, *n* (%)	44 (14)
Pancreatic tumor location, *n* (%)	
Head	165 (52)
Body	94 (30)
Tail	59 (19)
Tumor size (mm), *n* (%)	32 (1 – 98)
<20	41 (13)
20 – 40	181 (57)
>40	96 (30)
Histology, *n* (%)	
Adenocarcinoma	271 (85)
Others	47 (15)
Unknown	37 (12)
Site of metastatic disease, *n* (%)	
Liver	154 (48)
Peritoneum	62 (19)
Lung	39 (12)
Number of metastatic sites, *n* (%)	
≥2	97 (31)
Ascites, n (%)	62 (19)
Albumin (g/dL), *n* (%)	
>4	32 (10)
3 – 4	181 (57)
<3	105 (33)
LDH (U/L), *n* (%)	
<240	269 (85)
240 – 360	105 (33)
>360	8 (3)
CRP (mg/dL), *n* (%)	
<0.3	158 (50)
0.3 – 3.0	117 (37)
>3.0	43 (14)
CA19–9 (U/mL), *n* (%)	
<37	76 (24)
37 – 370	72 (23)
>370	170 (53)
AJCC TNM stage, *n* (%)	
III	63 (20)
IV	255 (80)
First line regimen, *n* (%)	
GnP	200 (63)
FFX	118 (37)

*Abbreviations: ECOG PS* Eastern Cooperative Oncology Group performance status, *LDH* lactate dehydrogenase, *CRP* C–reactive protein, *CA19–9* carbohydrate antigen 19–9, *AJCC* American Joint Committee on Cancer, *GnP* gemcitabine plus nab–paclitaxel, *FFX* FOLFIRINOX

The results of the univariate and multivariate analysis are listed in Table 2. The univariate analyses identified higher ECOG PS scores, presence of liver metastasis, more than two sites of metastatic disease, presence of ascites, serum albumin level less than 3.0 g/dL, elevated serum LDH, elevated serum CRP, serum CA19–9 level greater than 370 U/mL, and AJCC TNM stage IV as being significantly associated with shorter overall survival times. Multivariate analysis identified that ECOG PS, presence/absence of liver metastasis, serum LDH, serum CRP, and serum CA19–9 as independent predictors of the overall survival time.

**Table 2:** Univariate and Multivariate Cox proportional hazards models to predict survival in patients with unresectable pancreatic cancer

Variables	Univariate analysis			Multivariate analysis		
	HR	95%CI	*P*–value	HR	95%CI	*P*–value
Age						
<60	reference					
60–70	0.99	0.74 – 1.35	0.998	1.07	0.77 – 1.50	0.674
>70	0.99	0.70 – 1.39	0.936	1.08	0.73 – 1.59	0.710
Sex						
F	reference					
M	0.92	0.71 – 1.19	0.517	0.96	0.71 – 1.28	0.766
ECOG PS						
0	reference					
1	1.54	1.16 – 2.04	0.003	1.43	1.03 – 1.93	0.033
2 or more	2.22	1.30 – 3.79	0.003	2.52	1.34 – 4.71	0.004
Body mass index						
<22	reference					
≥22	1.01	0.77 – 1.30	0.969	0.98	0.73 – 1.31	0.880
Tumor resection						
no	reference					
yes	0.73	0.50 – 1.07	0.103	0.71	0.45 – 1.12	0.142
Pancreatic tumor location						
Head	reference					
Body	1.04	0.78 – 1.39	0.800	0.89	0.63 – 1.25	0.496
Tail	1.14	0.81 – 1.61	0.461	0.72	0.47 – 1.10	0.133
Tumor size						
<20	reference					
20 – 40	0.99	0.67 – 1.47	0.979	0.94	0.60 – 1.47	0.775
>40	1.39	0.91 – 2.11	0.125	0.88	0.54 – 1.43	0.605
Histology						
Adenocarcinoma	reference					
Others	1.09	0.75 – 1.56	0.656	1.26	0.83 – 1.90	0.280
Liver metastasis						
No	reference					
Yes	2.08	1.61 – 2.69	< 0.001	1.85	1.26 – 2.73	0.002
Peritoneal metastasis						
No	reference					
Yes	0.95	0.68 – 1.31	0.742	0.91	0.57 – 1.44	0.674
Lung metastasis						
No	reference					
Yes	1.39	0.95 – 1.31	0.09	1.40	0.86 – 2.29	0.176
Number of metastasis						
0 or 1	reference					
2 or more	1.59	1.21 – 2.09	< 0.001	1.17	0.80 – 1.71	0.422
Ascites						
No	reference					
Yes	1.52	1.12 – 2.05	0.007	1.37	0.93 – 2.00	0.112
Albumin (g/dL), n (%)						
>4	reference					
3 – 4	0.82	0.54 – 1.24	0.349	1.45	0.88 – 2.37	0.144
<3	0.56	0.36 – 0.88	0.012	1.29	0.72 – 2.27	0.399
LDH (U/L), n (%)						
<240	reference					
240 – 360	1.91	1.32 – 2.77	< 0.001	1.51	0.97 – 2.33	0.065
>360	2.90	1.36 – 6.19	0.006	2.88	1.35 – 6.82	0.007
CRP (mg/dL), n (%)						
<0.3	reference					
0.3–3.0	1.45	1.09 – 1.92	0.010	1.12	0.81 – 1.55	0.503
>3.0	3.04	2.09 – 4.43	< 0.001	2.04	1.23 – 3.36	0.005
CA19–9 (U/mL), n (%)						
<37	reference					
37 – 370	1.24	0.84 – 1.84	0.285	1.18	0.77 – 1.80	0.441
>370	1.91	1.36 – 2.68	< 0.001	1.45	1.01 – 2.07	0.043
AJCC TNM stage, n (%)						
III	reference					
IV	1.73	1.24 – 2.44	0.001	1.14	0.70 – 1.86	0.606
First line regimen, n (%)						
FFX	reference					
GnP	0.86	0.66 – 1.11	0.249	0.99	0.72 – 1.36	0.942

*Abbreviations*: *HR* Hazard ratio, *CI* Confidence interval, *ECOG PS* Eastern Cooperative Oncology Group performance status, *LDH* lactate dehydrogenase, *CRP* C–reactive protein, *CA19–9* carbohydrate antigen 19–9, *AJCC* American Joint Committee on Cancer, *GnP* gemcitabine plus nab–paclitaxel, *FFX* FOLFIRINOX

**Fig. 2:**
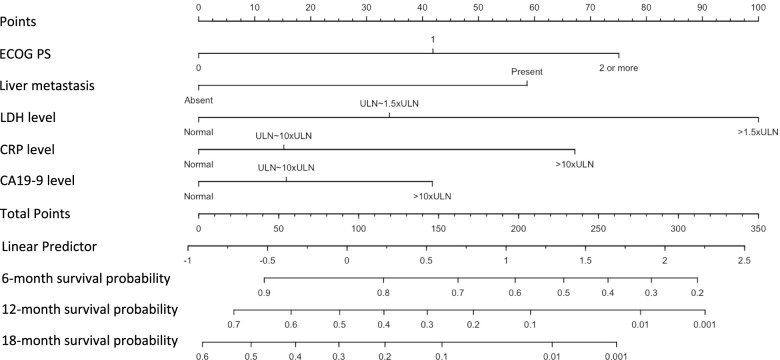
The prognostic nomogram for predicting the 6–, 12–, and 18–month survivals.

The prognostic nomogram integrating all the significant independent predictors of the overall survival identified by the multivariate analysis is shown in Fig. 2. The values of the C–index (bootstrapping 95% confidence intervals [CIs]) of the prognostic nomogram for overall survival prediction were 0.77 (0.73–0.81), 0.72 (0.67–0.76), and 0.70 (0.65–0.75) for 6–, 12–, and 18–month survival, respectively. These values were statistically significantly higher for all the points examined, as compared to the values for the AJCC TNM staging system (all *P* values<0.01) (Table 3, Fig. S1).

**Table 3:** Comparison of *t*–AUC between Nomogram and AJCC Staging system

	*t*–AUC (Nomogram)	*t*–AUC (AJCC Stage)	*P*–value
6–month	0.766	0.546	< 0.001
12–month	0.715	0.554	< 0.001
18–month	0.703	0.557	< 0.001

*Abbreviations*: *AUC* Area under the curve, *AJCC* American Joint Committee on Cancer

The calibration plot for the probability of survival at 6, 12, and 18 months showed optimal agreement between the predictions according to the nomogram and the actual observations (Fig. S2). The mean absolute errors between the observed and predicted probabilities were <0.01, 0.03 and 0.04 for 6–, 12–, and 18–month survival, respectively. The errors for 90% of the study population were within 0.01, 0.02 and 0.08, respectively.

**Fig. 3:**
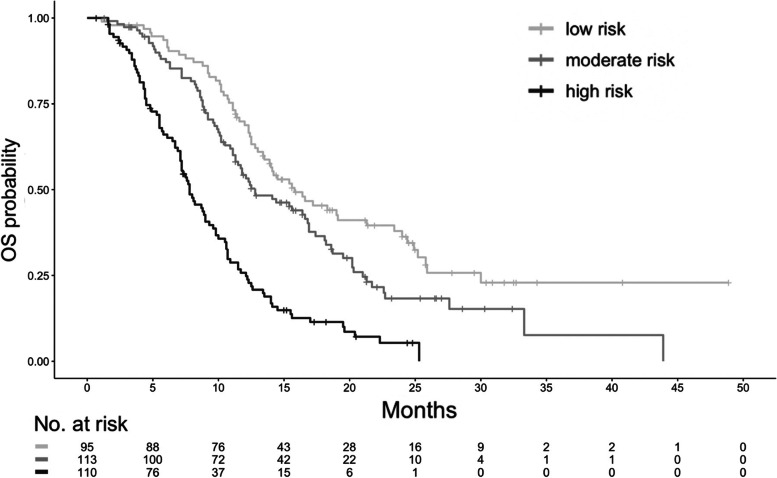
The median survival times in the low–, moderate–, and high–risk groups were 15.8 months (reference), 12.8 months (HR, 1.44; 95% CI, 1.03–2.01; *P*=0.03), and 7.8 months (HR, 3.34; 95% CI, 2.40–4.64; *P*<0.01), respectively.

Risk stratification by using the tertiles of the total nomogram points yielded clear separations among the survival curves. The median survival times in the low– (total nomogram points <56), moderate– (total nomogram points 56–115), and high– (total nomogram points ≥115) risk groups were 15.8 months (reference), 12.8 months (Hazard ratio [HR], 1.44; 95% CI, 1.03–2.01; *P*=0.03), and 7.8 months (HR, 3.34; 95% CI, 2.40–4.64; *P*<0.01), respectively (Fig. 3).

## Discussion

In this study, we developed a convenient prognostic nomogram based on five independent prognostic variables (ECOG PS, presence/absence of liver metastasis, serum LDH, serum CRP, and serum CA19–9) which could accurately predict survival in patients with unresectable pancreatic cancer undergoing treatment with GnP or FOLFIRINOX. Currently, the AJCC TNM staging system is the most widely used prognostic tool for patients with cancer, including pancreatic cancer. However, this staging system has a few limitations in regard to the analysis of survival. Importantly, it focuses only on tumor characteristics, while the importance also of patient–related factors in determining the disease outcomes in cancer patients has come to be increasingly recognized in recent years [17] Thus, we were prompted to develop a more accurate prognostic tool, and the nomogram that we have developed is an inexpensive tool based on easily determined variables, including both patient and tumor characteristics; it is expected to be a helpful tool for clinicians engaged in the treatment of unresectable pancreatic cancer patients.

ECOG PS is recognized as one of the most important prognostic factors in patients with a variety of cancers [18, 19], and as in the present study, several previous studies have also reported ECOG PS as an independent prognostic factor in patients with pancreatic cancer [20, 21]. We demonstrated herein that the patient prognosis became poorer as the ECOG PS score increased.

Presence of liver metastasis has been reported as an important predictor of survival in patients with various cancers [4, 22], and the MPACT trial showed that the presence of liver metastasis is an important predictor of survival also in patients with pancreatic cancer [4]. Among the distant metastases, including those to the liver, lung and peritoneum, it is unclear why only the presence of liver metastasis was associated with a poor prognosis in our study. Liver metastasis is associated with activation of hepatic stellate cells, which are key components of the hepatic tumor microenvironment and can acquire chemoresistance [23, 24]. Another possible explanation is that patients with liver metastasis could eventually develop jaundice or hepatic coma with increase in the number of metastatic tumors, which would make it difficult to continue with effective systemic chemotherapy, and potentially result in a fatal outcome.

An elevated serum LDH level in pancreatic cancer patients has been recognized as an indicator of tumor aggressiveness, tumor burden, and poor outcome [25], and has also been associated with chemoresistance to several anticancer–drugs, including paclitaxel and gemcitabine [26]. These phenomena might be explained by tumor hypoxia, which promotes the growth of immature and highly permeable blood vessels that drive the abnormal growth and metastatic behavior of pancreatic cancer and facilitate the passage of tumor cells into the circulation [27]. Actually, serum level of LDH significantly increases in hypoxic condition, and serves as an indirect marker of tumor hypoxia [25]. For these reasons, the results of our study, consistent with previous reports, also suggested that an elevated serum LDH level might be associated with a poor prognosis [25, 28].

An elevated serum CRP level has also been demonstrated to be an independent prognostic factor in patients with various types of cancers [29]. Proinflammatory cytokines, such as interleukin–6 (IL–6), interleukin–1, and tumor necrosis factor–alpha, are secreted by monocytes or macrophages under inflammatory conditions and cancer [30]. Serum concentrations of IL–6 and CRP are known to be positively correlated with each other, and recent evidence suggests that IL–6 also affects the rate of cancer progression [31]. Furthermore, there is also evidence to suggest that these inflammatory cytokines play important roles in the genesis of cancer–associated cachexia, which shortens the survival time in patients with advanced pancreatic cancer [32, 33].

Serum CA19–9 is the only biomarker that the National Comprehensive Cancer Network guidelines for pancreatic cancer suggest is useful as a prognostic marker in patients receiving chemotherapy [34]. One prospective study has reported the possible usefulness of serum CA19–9 as a prognostic biomarker in patients with advanced pancreatic cancer [35], and another prospective study showed that a decrease of the serum CA19–9 level during chemotherapy is predictive of a longer survival time in patients with advanced pancreatic cancer [36].

Our prognostic nomogram was created based on the above theoretical background of the above–mentioned independent prognostic factors. Then, we verified the nomogram by determining the values of the C–index and *t*–AUC, and constructing a calibration plot and Kaplan–Meier curves for the three risk categories. The values of the C–index of the nomogram for 6–, 12–, and 18–month survival were all more than 0.7, indicating a good match between the predicted and actual survival. Calibration and validation using the bootstrapping method also indicated satisfactory performance of the nomogram. In addition, total nomogram points can also be useful for predicting the survival, and Kaplan–Meier curves constructed using tertiles of the total nomogram points showed clear separations among the survival curves. Moreover, our nomogram provided better predictive performance for overall survival as compared to the AJCC TNM staging system using *t*–AUC. Notably, our nomogram was not only based on the real–world data of patients treated with GnP or FOLFIRINOX, but also constructed using conventional variables which can easily be obtained at any medical institution in daily practice. Compared with previous nomogram in pancreatic cancer, our nomogram was created using larger cohort of 14 institutions, which could improve the accuracy of the model. In addition, our nomogram can predict prognosis not only at 6–month but also at 12– and 18–month [37]. Thus, this nomogram can be helpful to clinicians for making appropriate clinical decisions in daily practice. Furthermore, another benefit of this prognostic nomogram includes the possibility of selecting patients who are fit for clinical trials.

This study had several limitations. Firstly, it was a non–randomized, retrospective study, which could introduce selection bias, with a smaller number of patients as compared to previous studies [38]. Thus, we were unable to include several patient data, such as weight loss, quality of life, and screening status before the diagnosis, which were not fully documented in the hospital records. To compensate the small number of cases, we selected the bootstrapping method which is statistically established as adjusting the optimism of the model [39], and there are several studies which also adopted this method [10-12 ] . We also consider it’s necessary to extend the number of patients and validate the results including other regimens, so we are going to collect patients’ data prospectively and analyze in the future. The second limitation was that the study lacked cross–validation so as that it would be difficult to generalize our results to other cohorts. However, we developed the nomogram using a spatiotemporally heterogeneous population recruited from multiple centers, which could contribute to improving the validity of this model. The third limitation was that our study focused on specific race and geographical location. Since this study was focused only on Japanese, it might be difficult to apply the results to other race or geographical regions. However, there are some similarities between our results and those of other regions. Previous studies from other regions reported that CA19–9, performance states and liver metastases are associated with survival as we showed [20, 37, 40]. Prognostic factors such as performance states and tumor marker were also identified in other cancers [41, 42]. Although it is a matter of speculation, these factors might be common regardless of race, regions or type of cancers. Finally, some patients were only clinically diagnosed as having pancreatic cancer, without histological confirmation. These indicate that some patients in the real–world situation have no choice, but to receive systemic chemotherapy without histological evidence for various reasons, including those related to the patients themselves and/or to the facilities that they seek treatment at.

In conclusion, our prognostic nomogram might be a convenient and inexpensive tool for accurate prediction of the prognosis in Japanese patients with unresectable pancreatic cancer undergoing treatment with GnP or FOLFIRINOX, and will help clinicians in selecting appropriate therapeutic strategies for individualized management.

## Supplementary Information

**Additional file 1: Figure S1.** The C–indices were statistically significantly higher for all the points examined, as compared to those for the AJCC TNM staging system.

**Additional file 2: Figure S2.** The calibration plot for the probability of survival at 6–, 12–, and 18–months showed optimal agreement between the predictions according to the nomogram and the actual observations.

## Data Availability

All data generated or analyzed in this article are stored in a secured research database. They are not publicly available, however, are available through the corresponding author on reasonable request.

## Abbreviations

GnP: gemcitabine plus nab–paclitaxel
C–index: concordance index
AJCC: American Joint Committee on Cancer
ECOG PS: Eastern Cooperative Oncology Group performance status
LDH: lactate dehydrogenase
CRP: C–reactive protein
CEA: carcinoembryonic antigen
CA19–9: carbohydrate antigen 19–9
*t*–AUC: time–dependent area under the curve
ROC: receiver operating characteristic
CI: confidence interval
HR: hazard ratio
IL–6: interleukin–6
