# Supplementary figures and images for: Prognostic nomogram for patients with unresectable pancreatic cancer treated with gemcitabine plus nab–paclitaxel or FOLFIRINOX: A post–hoc analysis of a multicenter retrospective study in Japan (NAPOLEON study)
Source: BMC Cancer. 2022 Jan 3;22:19. doi: 10.1186/s12885-021-09139-y (PMC8722136; doi:10.1186/s12885-021-09139-y)

6 months

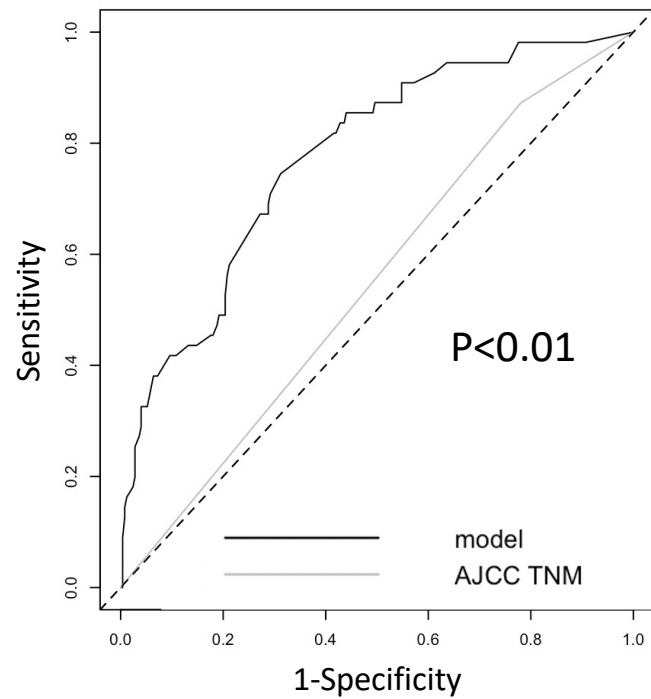

12 months

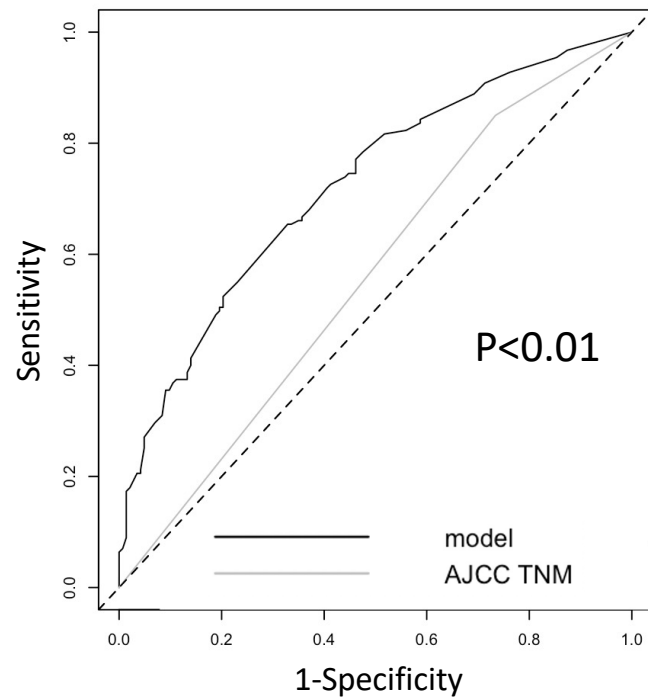

18 months

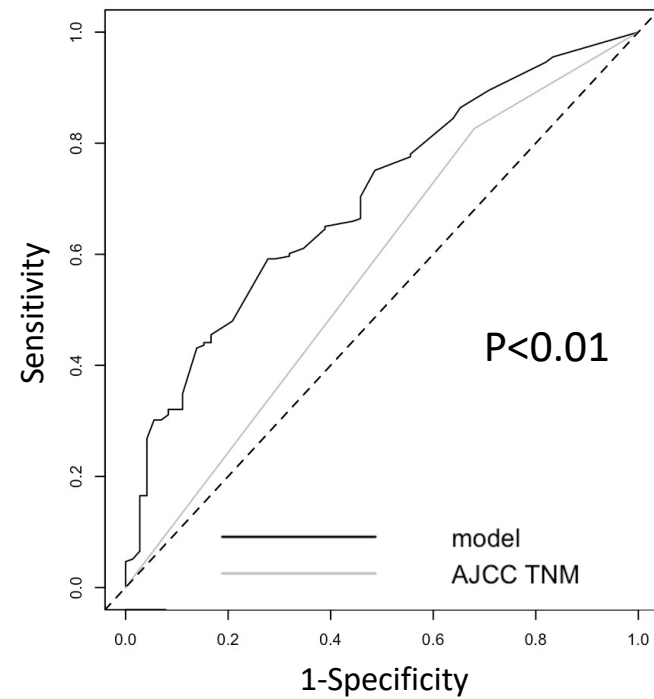

Supplement: Supplementary file 1 — Additional file 1: Figure S1. The C–indices were statistically significantly higher for all the points examined, as compared to those for the AJCC TNM staging system. [file 12885_2021_9139_MOESM1_ESM.pdf]

**6-month survival**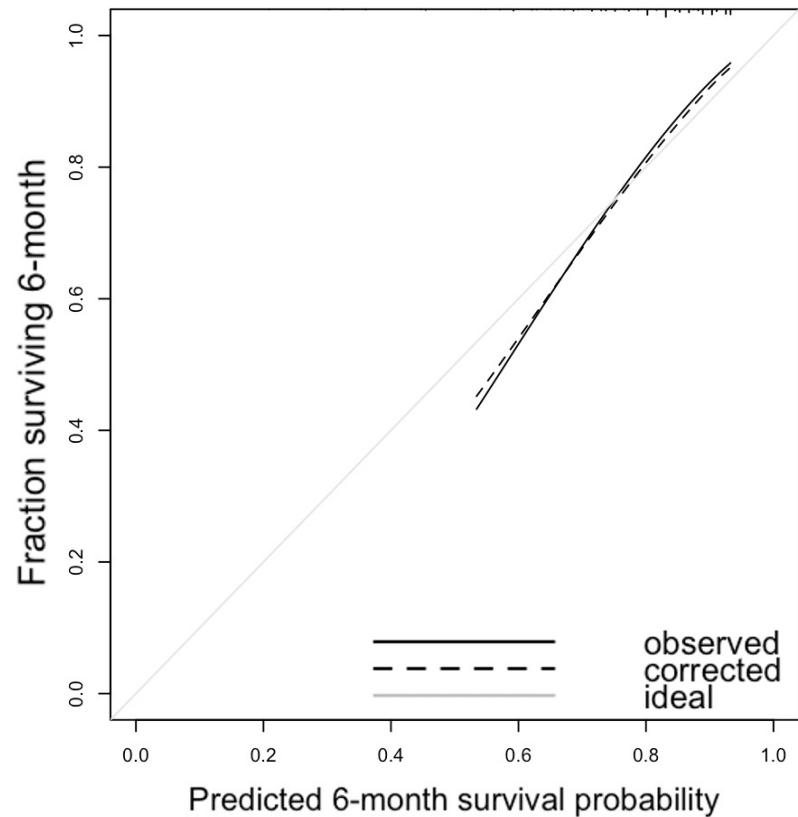**12-month survival**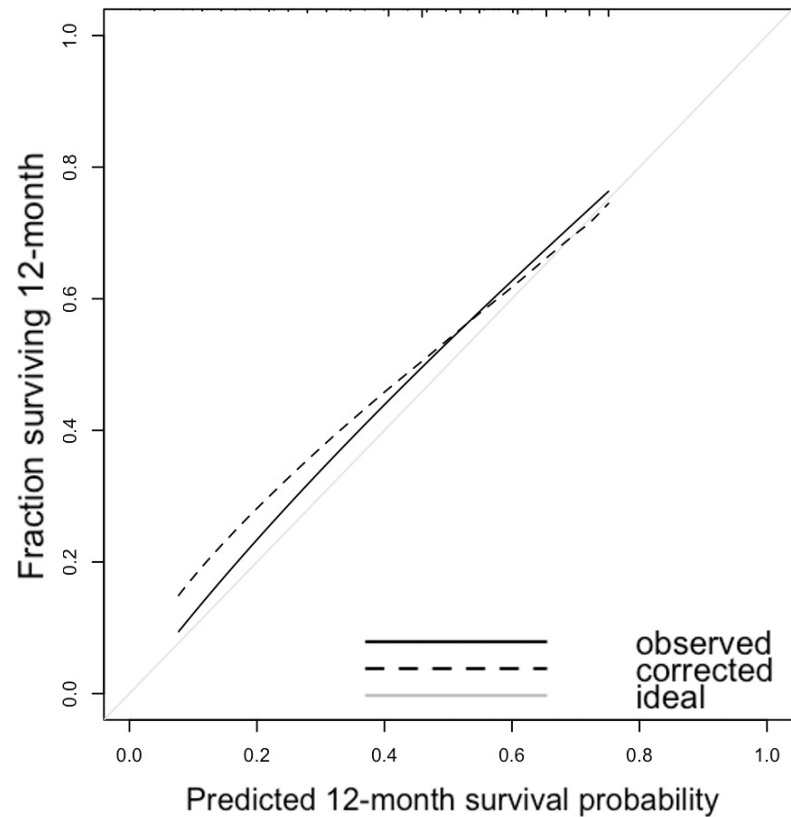**18-month survival**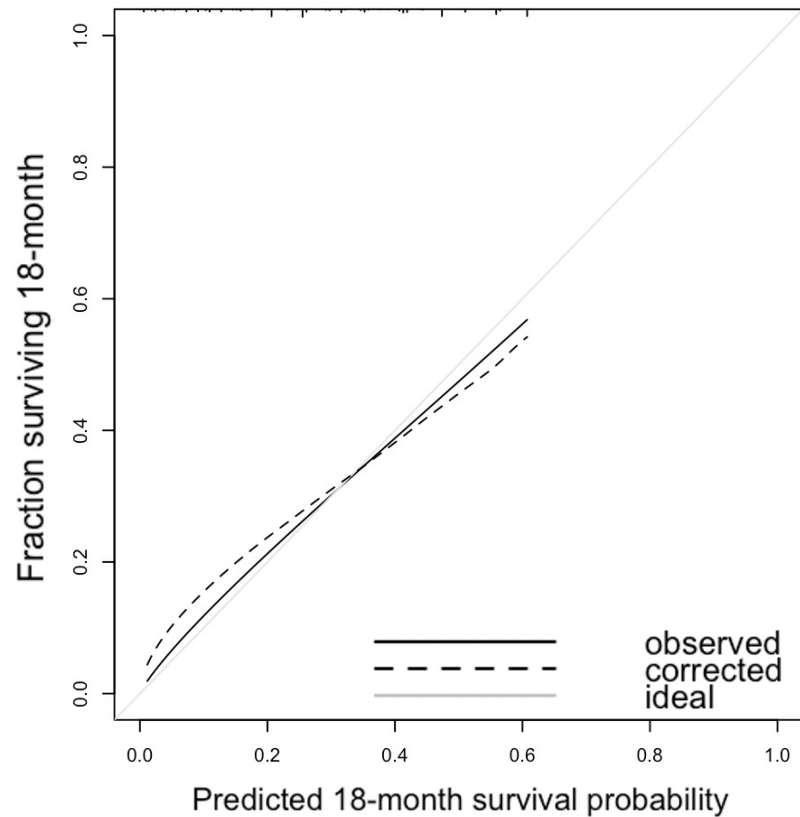

Supplement: Supplementary file 2 — Additional file 2: Figure S2. The calibration plot for the probability of survival at 6–, 12–, and 18–months showed optimal agreement between the predictions according to the nomogram and the actual observations. [file 12885_2021_9139_MOESM2_ESM.pdf]
